# Research progress on exercise-induced executive function improvements in older adults: insights from functional near-infrared spectroscopy

**DOI:** 10.3389/fpsyg.2026.1675737

**Published:** 2026-02-25

**Authors:** Zhidong Cai, Sen Li

**Affiliations:** 1 Department of Physical Education, Suzhou University of Science and Technology, Suzhou, China; 2 School of Physical Education and Health, Shanghai Lixin University of Accounting and Finance, Shanghai, China

**Keywords:** executive function, exercise interventions, functional near-infrared spectroscopy, neurovascular coupling, older adults

## Abstract

Functional near-infrared spectroscopy (fNIRS) has emerged as a promising technique in motor cognitive neuroscience and has become an important neuroimaging tool for the study of motor cognition. This review synthesizes evidence from fNIRS studies to elucidate the neural mechanisms that underlie exercise-induced improvements in executive function in older adults. A systematic search was conducted across six electronic databases from inception to March 20, 2025, and 27 relevant articles were included. These studies were systematically reviewed to examine the neural mechanisms by which exercise improves executive function in older adults along five dimensions: (1) resting-state brain activity; (2) task-evoked brain activity during executive function tasks; (3) acute exercise-induced immediate improvement in brain activity; (4) sustained effects on brain activity following acute exercise; and (5) long-term enhancements in brain activity after regular physical exercise. The results showed that a decrease in cerebral oxygenation accompanied brain aging, weakened hemodynamic oscillations, and abnormal resting-state functional coupling. A two-stage neural compensation model may underlie the exercise intervention aimed at improving executive function in older adults. Acute exercise can temporarily improve executive function by expanding the “resource pool” to increase neural resources and enhance prefrontal cortical hemodynamic activity and recruitment of neural resources. Chronic exercise achieves structural–functional optimization and efficient use of neural resources through the accumulation effect of repeated acute exercise stimulation, thereby continuously improving executive function. Therefore, we suggest that future studies should conduct large-scale RCTs using multimodal neuroimaging methods combining ERP, fMRI, and fNIRS. This will compensate for the shortcomings of fNIRS and provide a deeper understanding of how exercise remodels brain networks, thereby establishing a theoretical basis for precision interventions targeting brain aging.

## Introduction

Global demographic shifts reveal accelerated population aging, with the ≥60 age cohort expanding at 3% annually (UN DESA, 2017). This epidemiological transition poses significant public health challenges, particularly regarding age-related cognitive decline. Chinese epidemiological data demonstrate age-stratified prevalence patterns: mild cognitive impairment (MCI) rates escalate from 11.83 to 32.46%, while dementia prevalence rises from 2.89 to 31.23% across successive age decades (Jia et al., 2020). Executive function (EF) is a meta-level top-down psychological process involving conscious supervision, monitoring, and control of non-automatic behaviors to achieve the goal of autonomous choice. Inhibition, working memory, and shifting are three core components (Miyake et al., 2000, Diamond, 2020). Specifically, inhibition refers to the ability to intentionally suppress or control dominant stimuli and filter out irrelevant cues (Logan, 1994, Zhan et al., 2020); working memory refers to the ability to temporarily retain and process relevant information in mind (Baddeley, 1992, Ku, 2019); shifting is defined as the ability to flexibly switch between mental sets, operations, or conceptual representations (Monsell, 1996, Kofler et al., 2019). The frontal lobe of the brain largely controls EF, and its decline is an important factor in age-related cognitive decline, indicating that older adults have a higher risk of developing mild cognitive impairment and dementia later in life. The decline of executive function has a serious impact on the quality of life of the elderly, posing a heavy burden on their families and society. Maintaining brain health, preventing or delaying cognitive decline, and improving the quality of life in old age has become an urgent issue for aging societies.

Physical exercise is characterized as a planned, structured, and repetitive physical activity aimed at improving or maintaining one’s physical fitness. It encompasses cardiorespiratory (aerobic) exercises, resistance exercises, flexibility exercises, and neuromotor training (Garber et al., 2011). Although increasing evidence suggests that physical exercise can enhance executive function in older adults (Chen et al., 2020, Singh et al., 2025), the neurobiological mechanisms underlying exercise-induced improvements remain unclear. The advent of neuroimaging modalities, especially portable technologies like functional near-infrared spectroscopy (fNIRS), has revolutionized mechanistic investigations of exercise-cognition interactions (Herold et al., 2020; Herold et al., 2018). Cerebral metabolic demands and vascular responses form the neurovascular basis of cognitive processing, with prefrontal oxygenation levels strongly predicting executive performance (Byun et al., 2014). Experimental evidence demonstrates that acute exercise bouts transiently enhance prefrontal oxygenated hemoglobin concentrations during cognitive tasks (Hyodo et al., 2012), while longitudinal exercise engagement predicts increased resting-state oxygenated hemoglobin (oxyHb) levels in the left dorsolateral prefrontal cortex—a key node for executive function (Suhr and Chelberg, 2013).

Although numerous studies have employed fNIRS to investigate the beneficial effects of physical exercise on cognitive function in older adults, the findings exhibit inconsistencies. Consequently, the mechanisms underlying exercise-induced brain improvement remain unclear. This review aims to synthesize the literatures utilizing fNIRS in researches on physical exercise for mitigating cognitive aging. Specifically, it seeks to examine the impact of physical exercise on cerebral blood oxygenation and brain activation levels, and to explore potential mechanisms through which physical exercise ameliorates age-related cognitive decline. This synthesis is expected to facilitate the improvement of clinical diagnostics and provide valuable insights for the formulation of interventions aimed at executive function in the elderly population.

## Methods

### Search strategy

We searched six electronic databases [PubMed, Cochrane Library, Web of Science, Scopus, Wanfang Data, and China National Knowledge Infrastructure (CNKI)] from inception to March 20, 2025. The research strategy used for this review was as follows:

#1 acute exercise OR a single bout exercise OR physical exercise OR physical activity OR aerobic exercise OR resistance exercise OR strength exercise OR stretching OR mind-body exercise OR flexibility exercise OR coordinative training OR multicomponent exercise

#2 cognition OR executive function OR executive control OR inhibition OR working memory OR shifting

#3 old people OR elderly OR old age OR the aged OR senior citizen OR older adults

#4 functional near-infrared spectroscopy OR fNIRS OR NIRS

#5 #1 AND #2 AND #3 AND #4

### Inclusion and exclusion criteria

The inclusion criteria were as follows: (1) the participants were older adults; (2) the intervention was an exercise program; and (3) the outcome measures included both executive functions (at least one of inhibition, working memory, and shifting) and cerebral oxygenation. The exclusion criteria included two main aspects: (1) the intervention protocol contained confounding factors associated with non-exercise interventions; (2) the study type was a meta-analysis, review, case report, conference paper, editorial, or other nonoriginal research article; and (3) data could not be extracted from the study even after contacting the authors.

### Data collection and extraction process

Two researchers independently screened the records based on predefined inclusion and exclusion criteria. In cases of disagreement between the two reviewers, a third researcher resolved the discrepancy by reviewing the full text. The final included studies were subjected to a strict screening process. The extracted data recorded in the database included: (a) first author and year of publication; (b) sample size, age, gender, and other relevant characteristics of the study population; (c) experimental protocols; (d) cerebral oxygenation; and (e) primary outcomes of the study.

## Results

### Characteristics of included studies

**Figure 1:**
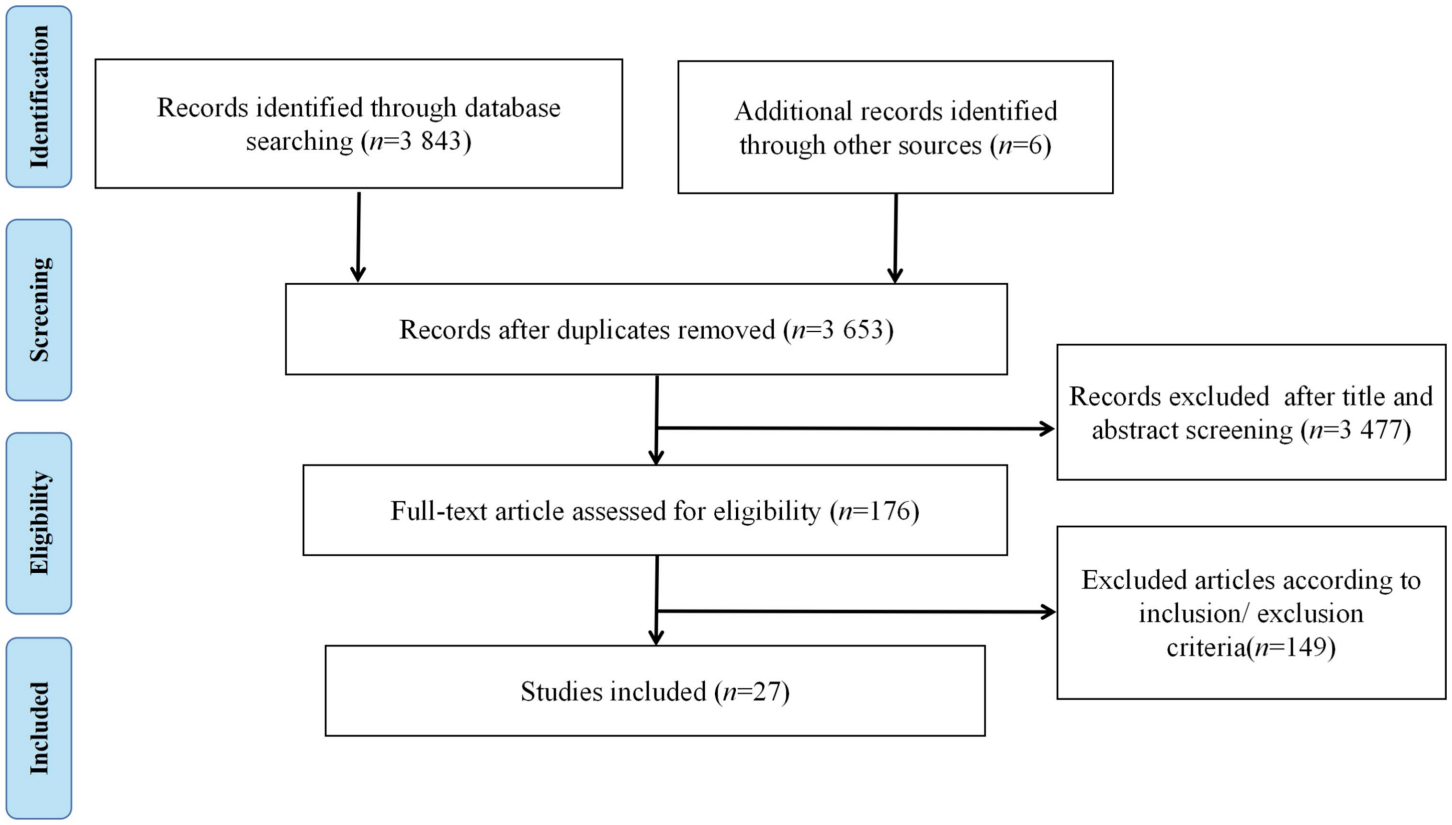
The flow diagram of study selection.

A total of 3,849 relevant articles were retrieved from the literature. After removing 214 duplicates and screening by title and abstract, 125 articles remained. Finally, 27 studies were included (Figure 1). Among them, 9 studies investigated changes in cerebral hemoglobin concentration at rest in older adults (Table 1), 12 explored changes in cerebral hemoglobin concentration during executive function tasks after or during acute exercise (Table 1), and 6 investigated the effects of chronic exercise intervention on cerebral hemoglobin concentration during executive function tasks in older adults (Table 2).

**Table 1:** A summary of fNIRS studies on resting and acute exercise.

Study ID	Sample characteristics	fNIRS parameters	Experimental protocol	Main findings
Amiri et al. (2014)	Young: *n* = 23 (8 M, 15F); M = 23.4, SD = 2.7, range: 20–35 Old: *n* = 23 (8 M, 15F); M = 69.6, SD = 4.1, range: 65–75	Device 1: 25 Hz; 58 bilateral frontotemporal channels (TechEn CW6) Device 2: 10-, 15-, 25-, and 30-mm separation; 4 forehead channels	Resting	Older adults exhibited reduced resting (oxyHb) and oxygen saturation in the bilateral PFC compared to younger adults. Anatomical magnetic resonance imaging and arterial spin label data also revealed lower resting blood flow in both frontal and temporal lobes compared to younger adults.
Tan et al. (2016)	Young: *n* = 40 (27 M, 13F); M = 24.5, SD = 1.7, range: 20–30 Old: *n* = 43 (23 M, 20F); M = 69.6, SD = 8.4, range: > 60	10 Hz; 2 bilateral forehead channels (TSAH-200; Tsinghua University, China)	Sitting (20 min)	Older adults exhibited significantly reduced wavelet amplitude within the 0.052–0.145 Hz and 0.0095–0.021 Hz frequency intervals in both left and right PFC regions compared to younger adults. Older adults demonstrated decreased wavelet phase coherence specifically within the 0.6–2 Hz and 0.052–0.145 Hz frequency ranges relative to their younger counterparts.
Wang et al. (2016)	Young: *n* = 22 (14 M, 8F); M = 24.4, SD = 1.6 Old: *n* = 39 (23 M, 16F); M = 70.5, SD = 7.7	10 Hz; 2 bilateral forehead (TH200; Tsinghua University, China) and 2 bilateral sensorimotor cortex (OXYMON MK III; Artinis Medical Systems, B. V., Netherlands) channels	Sitting (20 min) followed by standing (10 min)	Older adults exhibited reduced interhemispheric connectivity within the [PFC and diminished PFC-sensorimotor cortex connectivity across all frequency bands (0.0095–2 Hz, excluding 0.052–0.145 Hz) relative to younger adults during both seated and standing postures]. Older adults demonstrated enhanced PFC-sensorimotor cortical connectivity specifically in the 0.052–0.145 Hz frequency range during standing compared to younger adults.
Huo et al. (2018)	Young: *n* = 19 (one excluded; 13 M, 7F); M = 24.1, SD = 1.9 Old: *n* = 17 (one excluded; 13 M, 5F); M = 69.4, SD = 9.6	3-cm separation; 10 Hz; 36 bilateral prefrontal, central, and occipital channels (NirScan Danyang Huichuang Medical Equipment CO. Ltd)	Sitting (15 min) followed by standing (10 min)	In older adults, the transition from sitting to standing enhanced the regulation of the motor cortex by the PFC and occipital lobe. Conversely, in younger adults, this posture change increased the regulation of the PFC and occipital lobe by the motor cortex. When seated, older adults exhibited decreased connectivity between the left and right PFC, as well as reduced connectivity from the prefrontal or occipital cortex to the motor cortex compared to their younger counterparts. However, when standing, they demonstrated increased connectivity from the motor cortex to the PFC relative to younger individuals.
Mukli et al. (2018)	Young: *n* = 24; M = 30.6, SD = 8.2, range: < 45 Old: *n* = 28; M = 60.5, SD = 12.0, range: ≥ 45	4-cm separation; 2 Hz; 1 forehead channel (NIRO 500; Hamamatsu Photonics, Hersching, Germany)	Sitting	Multifractal (total-Hb) analysis revealed increased long-range autocorrelation in vasogenic hemodynamics but decreased correlation in neural hemodynamic fluctuations among older adults compared to younger adults. Furthermore, (oxyHb) and (deoxyHb) fluctuations exhibited anticorrelated dynamics in younger adults, whereas this relationship was absent in the older cohort.
Viola et al. (2013)	NC: *n* = 10 (4 M, 6F); M = 69.5, SD = 6.8 aMCI: *n* = 21 (10 M, 11F); M = 70.2, SD = 7.3	4-cm separation; 4 bilateral frontal (F2–F8 and F1–F7) and temporal–parietal (P4–T4 and P3–T3) channels (T-NIRS EVO II)	Sitting	Patients with aMCI exhibited lower tissue oxygenation in the bilateral frontal and temporal–parietal regions compared to NC controls. This reduced tissue oxygenation was correlated with global cognitive functioning in patients with aMCI.
Liu et al. (2014)	NC: *n* = 21 (8 M, 13F); M = 67, SD = 7, range: 55–80 (*n* = 18 for fNIRS data) aMCI: *n* = 32 (13 M, 19F); M = 67, SD = 7, range: 55–80 (*n* = 26 for fNIRS data)	Forehead channels (NIRO-200NX, Hamamatsu Photonics)	Lying in the supine position (around 20 min)	fNIRS data revealed no group differences in PFC tissue oxygenation. Simultaneous measurements from fNIRS, color-coded duplex ultrasonography, a sphygmomanometer, and a pulse oximeter indicated that patients with aMCI exhibited lower normalized total cerebral blood flow and cerebral metabolic rates of oxygen but greater cerebrovascular resistance compared to NC controls.
Schroeter et al. (2003)	Young: *n* = 14 (7 M, 7F); M = 23.9; SD = 3.1, range: 19–29 Old: *n* = 14 (7 M, 7F); M = 65.3, SD = 2.9, range: 62–71	NIRO-300 spectrometer; Hamamatsu Photonics K. K., Hamamatsu, Japan	Resting with eyes closed (6 min)	Older adults exhibited a lower mean power spectral density of low-frequency (0.07–0.11 Hz) oscillations in both (oxyHb) and (deoxyHb) compared to younger adults during the resting state. No significant differences were observed between groups in the mean power spectral density of very-low-frequency (0.01–0.05 Hz) oscillations.
Laguë-Beauvais et al. (2013)	Young: *n* = 21 (9 M, 12F); M = 24.4, SD = 4.5, range: 19–36 Old: *n* = 19 (3 M, 16F); M = 64.0, SD = 3.4, range: 59–69	2.8-cm separation; 10 Hz (after downsampling); 26 bilateral prefrontal channels (CW5; TechEn Inc., Milford, MA)	Modified Stroop [neutral, congruent, interference, and switching (i.e., word reading in 25% of the trials) conditions; inter-trial intervals ranging from 8–16 s]	While task-switching elicited activation in isolated portions of the bilateral anterolateral PFC in younger adults, it elicited widespread activation in the bilateral anterolateral and posterolateral PFC in older adults.
Agbangla et al. (2019)	Young: *n* = 19 (17 M, 2F); M = 19.7, SD = 1, range: 18–22 Old: *n* = 37 (15 M, 22F); M = 69.0, SD = 4.7, range: 60–77 (higher-fit: n = 21; lower-fit: n = 16)	3.5-cm separation 10 Hz; 8 bilateral dorsolateral and ventrolateral PFC channels (OxyMon MkIII; Artinis Medical Systems BV, Zetten, Netherlands)	Letter *n*-back (0-, 1-, 2-, 3-back conditions; 150-s active task period; 30% targets)	Older adults exhibited greater WM-related increases in bilateral frontal (oxyHb) compared to younger adults during the 1-back task, but not during the 2- and 3-back tasks. Among older adults, those with higher fitness demonstrated a larger WM-related increase in (oxyHb) and a more pronounced decrease in (deoxyHb) in the left PFC than their lower-fit counterparts. Furthermore, 3-back sensitivity was positively correlated with changes in left PFC (oxyHb) specifically within the higher-fit older adult group.
	Young: *n* = 17 (7 M, 10F); M = 25.9, SD = 3.0, range: 21–32 Old: *n* = 17 (6 M, 11F); M = 70.7, SD = 5.2, range: 64–81	5-cm separation; 125 Hz; 2 bilateral forehead channels (Oxymon; Artinis Medical Systems, The Netherlands)	Letter *n*-back (0-, 1-, and 2-back conditions; 3.5 s per trial; 180 s per condition)	Whereas younger adults showed a right-lateralized increase in (oxyHb) in the PFC, older adults showed less lateralization of increased (oxyHb) during the 0-back and 1-back tasks. Whereas linear changes were observed for (oxyHb), (deoxyHb), and (total-Hb) during the late task period in younger adults, these signals plateaued or even decreased during the late task period in older adults during the 2-back task.
Yamanaka et al. (2014)	Young: *n* = 52 (30 M, 22F); M = 23.5, SD = 2.7 Old: *n* = 38 (17 M, 21F); M = 72.5, SD = 4.7, range: 64–83	3-cm separation; 10 Hz; 52 bilateral frontotemporal channels (ETG-4000; Hitachi Medical Corporation, Tokyo, Japan)	Spatial delayed match-to-sample (control and WM tasks under real or sham TMS; ten 42-s trials per condition; 17 s per trial)	Older adults exhibited greater increases in frontal (oxyHb) compared to younger adults across all conditions, with high-performing older adults demonstrating larger (oxyHb) increases than their low-performing counterparts. In younger adults, real transcranial magnetic stimulation at P3 induced decreases in frontal (oxyHb) during the WM task but increases in (oxyHb) during the control task, whereas stimulation at P4 produced an opposite pattern. For older adults, transcranial magnetic stimulation over either hemisphere of the parietal cortex did not modulate (oxyHb).
Causse et al. (2019)	Young: *n* = 18 (17 M, 1F); M = 21.0, SD = 1.6, range: 19–25 Middle-aged: *n* = 19 (17 M, 2F); M = 38.3, SD = 6.9, range: 30–48 Old: *n* = 24 (24 M, 0F); M = 62.3, SD = 6.6, range: 51–74	2.5-cm separation; 2 Hz; 16 forehead channels (Biopac fNIR 100)	Cambridge Neuropsychological Test Automated Battery Spatial WM (four difficulty levels) and One Touch Stockings of Cambridge (six difficulty levels)	Older adults exhibited a lower increase in (oxyHb) in the left lateral PFC compared to younger adults during the high-difficulty spatial WM task. Flying experience did not influence (oxyHb) levels in the left PFC among older adults. Age had no significant effect on (oxyHb) responses during the tower task.
Wijeakumar et al. (2017)	Young: *n* = 24; M = 25.4, SD = 4.3 Old: *n* = 24; M = 70.5, SD = 5.2	1-cm (4 channels) and 3-cm (36 channels) separation; 25 Hz; 40 bilateral frontal and parietal channels (TechEn CW6 system)	Change Detection task (‘simple’ color and ‘complex’ shape tasks; WM load varied from 1 to 3 items; 20 same and 20 different trials per load and run)	Older adults generally showed lower increases in (oxyHb) in posterior regions but larger increases in anterior regions than younger adults. Older adults exhibited decreased activity in the right intraparietal lobule and left supramarginal gyrus during the simple task, but increased activity in these regions during the complex task. During the complex task, older adults demonstrated increased activity in the right middle frontal gyrus at low load but decreased activity in this region at high load; no modulation of right middle frontal activity was observed during the simple task.
Harada et al. (2009)	Old: *n* = 15 (2 M, 13F); M = 63, SD = 4	3-cm separation; 5.26 Hz; 42 bilateral frontocentral channels (OMM-2001; Shimadzu, Kyoto, Japan)	Speed treadmill walking [target heart rate = (maximum heart rate - resting heart rate) * 30, 50%, or 70%, plus resting heart rate]	Older adults with low gait capacity had greater activation in the left PFC than older adults with high gait capacity at 70% walking intensity. Left PFC activation was positively associated with heart rate response, and supplementary motor area and medial sensorimotor cortex activation were positively associated with both cadence and velocity.
Lucas et al. (2012)	Young: *n* = 13 (7 M, 6F); M = 24, SD = 5 Old: *n* = 9 (4 M, 5F); M = 62, SD = 3	1 Hz; 1 right forehead channel (NIRO-200; Hamamatsu Photonics KK, Hamamatsu, Japan)	Color-word matching Stroop (simple and difficult conditions; task performance first at rest, then while cycling at 30 and 70% of the heart rate range)	Both younger and older adults exhibited comparable alterations in (oxyHb), (deoxyHb), and (total-Hb) levels during the Stroop task while cycling, irrespective of exercise intensity.
Belli et al. (2021)	Young: *n* = 24 (12 M, 12F); M = 22.7, SD = 1.30 Old: *n* = 25 (12 M, 13F); M = 67.37, SD = 5.31	3.5-cm separation; 10 Hz; 8 bilateral frontocentral channels (NIRO-200; Hamamatsu Photonics KK, Hamamatsu, Japan)	30 s of walking	Older adults exhibited a significant increase in activity within the left prefrontal cortex (PFC) when transitioning from their preferred to a faster walking pace, whereas young adults maintained consistent levels of PFC activity across these conditions.
Hyodo et al. (2012)	Old: *n* = 16 (13 M, 3F); M = 69.3, SD = 3.5, range: 64–74	3-cm separation; 10 Hz; 48 bilateral frontal channels (ETG-7000; Hitachi Medical Co., Kashiwa, Japan)	Matching Stroop (neutral and incongruent trials; 11–15 s per trial; 30 trials per condition and run)	An acute bout of moderate exercise resulted in a greater interference-induced increase in (oxyHb) in the right frontopolar area, which was associated with a larger exercise-induced reduction in Stroop reaction time interference than rest.
Wen et al. (2015)	Old: *n* = 15 (6 M, 9F); M = 58.7, SD = 7.2	3-cm separation; 10 Hz; 44 bilateral frontal channels (ETG4000; Hitachi Medical Co., Kashiwa, Japan)	Flanker (congruent task and incongruent trail, 30 trials per condition, 10 min moderate intensity cycling or rest)	The flanker interference in the oxyHb single experimental group was significantly higher than that of the control group in the left frontopolar area.
Jiang et al. (2019)	Old: *n* = 29; M = 65.8, SD = 4.3	3-cm separation; 10 Hz; 44 bilateral frontal channels (ETG4000; Hitachi Medical Co., Kashiwa, Japan)	Verbal fluency task (within 30 s, name as many species as possible,)	The Baduanjin group exhibited a greater increase in oxyHb within the prefrontal lobe brain regions compared to the control group.
Cai et al. (2023)	Old: *n* = 18 (9 M, 9F); M = 80, SD = 5.4	3-cm separation; 10 Hz; 24 bilateral frontal channels (Brite24, Artinis Medical Systems, Netherlands)	*N*-back (0, 1, 2 back, 40 min elasitc exercise or rest)	An increase in oxyHb and a decrease in deoxyHb were observed in the dorsolateral prefrontal and frontopolar regions.

PFC, prefrontal cortex; (deoxyHb) = deoxyhemoglobin concentration; (oxyHb) = oxyhemoglobin concentration; (total-Hb) = total hemoglobin concentration.

**Table 2:** A summary of fNIRS studies on the effects of chronic exercise intervention on executive function in older adults.

Study ID	Sample characteristics	fNIRS parameters	Experimental protocol	Activation tasks	Main findings
Wang et al. (2013)	Old: *n* = 12 (4 M, 8F); M = 64.25, SD = 3.14,range: 60–68	3-cm separation; 10 Hz; 24 bilateral frontal channels (Hitachi ETG-l00, Hitachi Medical Corporation, Tokyo, Japan)	T:60 min of Taichi I: NR D: 6 months F:1 time/week	Color-word matching Stroop	No significant alterations were noted in the prefrontal hemoglobin concentration during the task.
Yang et al. (2019)	Old: *n* = 26F; M = 66.12, SD = 3.81	3-cm separation; 10 Hz; 44 bilateral frontal channels (ETG4000; Hitachi Medical Co., Kashiwa, Japan)	T:45 min of Taichi I: NR D: 6 months F: 3 days per week	Flanker task (4 blocks, each block 30 trials)	Increase in (oxyHb) in the prefrontal cortex during the incongruent flankers after the Taichi intervention.
Eggenberger et al. (2016)	Old: *n* = 33 (12 M, 21F); M = 74.9, SD = 6.9, range: > 65	Four different separations; 10 Hz; 2 bilateral prefrontal channels (Oxiplex TS Tissue Spectrometer; ISS Inc., Champaign, IL, USA)	T: 30 min of dance I: NR D: 6 months Fr: 3 days per week	Treadmill walking (preferred or fast walking; 30-s walking periods; four blocks per condition)	The increase in bilateral prefrontal (oxyHb) during the acceleration phase of self-paced walking was generally lower after training. Dance training resulted in a smaller increase in left (oxyHb) compared to balance training during the deceleration phase of fast-paced walking.
Coetsee and Terblanche (2017)	Old: *n* = 72 (21 M, 46F); M = 62.7, SD = 5.7, range: > 65	4-cm separation; 5HZ; 2 bilateral prefrontal channels (NIRO 200NX, Hamamatsu, Japan	T: 30 min of RT or MCT or HIIT I: RT: 10RM; MCT:70–75% HRmax; HIIT:90-95%HRmax D: 16 weeks Fr: 3 days per week	Color-word matching Stroop (simple task: 24 trials, complex task: 24 trials)	At post-test, the control group showed increased brain activation with significantly higher relative (oxyHb) values during the naming Stroop condition compared to pre-test and a trend towards significance for their increased relative (oxyHb) in the complex condition. MCT and HIIT participants demonstrated decreased brain activation during the Stroop task, with MCT showing a significant increase in relative (deoxyHb) compared to pre-test during both naming and executive Stroop conditions.
Guo (2020)	Old: *n* = 39 F; M = 60, SD = 5.1	2.5-cm separation; 2 bilateral prefrontal channels (Glen Head, NY, USA)	T:40 min of Flexi-bar exercise I: moderate D: 8 weeks Fr:3 days per week	Color-word matching Stroop	The study revealed significant differences in activation levels between the left and right ventrolateral prefrontal cortex, as well as the right frontal pole region, during Stroop tasks following intervention.
Cai et al. (2024)	Old: *n* = 52 (20 M, 31 F); M = 83.6, SD = 6.8	3-cm separation; 10 Hz; 24 bilateral frontal channels (Brite24, Artinis Medical Systems, Netherlands)	T:40 min of elastic exercise I: low to moderate D: 16 weeks Fr:3 days per week	*N*-back (0, 1, 2 back)	Prefrontal activation was not significant under low memory load conditions. However, significant activation was observed in the bilateral ventrolateral prefrontal cortex, left dorsolateral prefrontal cortex, and left frontal pole regions under medium and high memory load conditions.

PFC, prefrontal cortex; (deoxyHb) = deoxyhemoglobin concentration; (oxyHb) = oxyhemoglobin concentration; (total-Hb) = total hemoglobin concentration. HIIT, high-intensity interval training; MCT, moderate-intensity continuous training; RT, resistance training; RM, repeated maximal; NR, no report; T, time; I, intensity; D, duration; Fr, frequency.

### Principles of functional near-infrared spectroscopy

**Figure 2:**
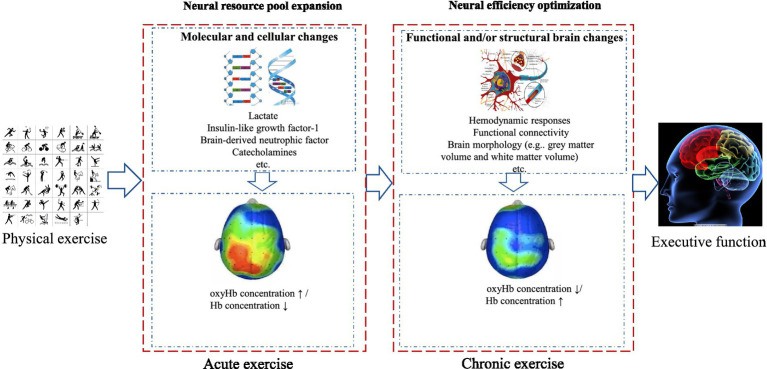
Dual-phase neural compensation model.

Functional near-infrared spectroscopy (fNIRS) is a non-invasive optical neuroimaging technique that operates on the principles of neurovascular coupling theory and optical spectroscopy. It provides an indirect measure of cerebral activity by monitoring variations in hemoglobin concentration within the brain. This method capitalizes on the unique absorption properties of chromophores, notably oxyhemoglobin (oxyHb) and deoxygenated hemoglobin (deoxyHb), to near-infrared light with wavelengths ranging from 600 to 900 nm. Specifically, wavelengths greater than 800 nm are predominantly absorbed by oxyHb, whereas those less than 800 nm are primarily absorbed by deoxyHb (Wyatt et al., 1986). Upon introducing near-infrared light into targeted cerebral areas, it traverses various tissue layers, including the scalp, skull, and cerebrospinal fluid, undergoing both absorption and scattering within the cerebral tissue. During this process, photon energy is transformed into the internal energy of the medium, while scattering results in photons deviating from their original path, elongating their travel distance. The remaining unabsorbed scattered light adopts a banana-shaped propagation pattern and is subsequently detected proximate to the light source using detectors. Based on the correlation between changes in chromophore concentration and light attenuation in brain tissue, combined with the modified Beer–Lambert law, it is possible to calculate the biochemical information carried by emergent light related to the optical properties of brain tissue after a series of absorption and scattering processes. This makes it possible to noninvasively quantify cortical OxyHb and DeoxyHb concentration changes, which can then be used to infer neuronal activity in the brain through the neurovascular coupling mechanism (as shown in Figure 2).

Neurovascular coupling is the fundamental principle underlying near-infrared brain imaging technology. Upon activation, cortical neurons prompt a localized surge in oxygen metabolic rate. The consumption of blood oxygen for energy production results in a temporary rise in deoxyHb concentration (Iadecola, 2017). Concurrently, to cater to the heightened energy requirements of these neurons, there is a swift increase in cerebral blood flow, causing an influx of oxyHb. This leads to an increased oxyHb concentration and a decreased deoxyHb concentration. Such distinct shifts are emblematic of the cerebral oxygenation dynamics induced by cortical activation. The seminal work by Jöbsis (1977) first demonstrated *in vivo* monitoring of cerebral oxygenation in feline and human models using NIRS. Subsequent technological advancements have established fNIRS as an essential tool in cognitive neuroscience research. Based on Scholkmann et al. (2014) classification, all included studies used continuous wave instruments.

### Characteristics of resting state cerebral oxygenation in older adults

Brain aging leads to reductions in brain volume and weight (Jernigan et al., 2001, Tari et al., 2025), accompanied by a decrease in cerebral blood flow (Vaidya et al., 2007, Zhu et al., 2024). The reduction in cerebral blood flow most significantly affects the frontal and parietal lobes, which constitute critical neural networks for cognitive function (Yuan and Raz, 2014); these changes are considered key contributors to age-related cognitive decline. Currently, there are two primary theories regarding brain activation mechanisms during cognitive aging. Based on neuroimaging evidence, Reuter-Lorenz and Cappell proposed the model of hemispheric asymmetry reduction in older adults (HAROLD), which suggests that older adults exhibit reduced lateralization of prefrontal activation during cognitive tasks, with the brain compensating for functional decline by recruiting bilateral prefrontal regions—an indicator of brain aging (Cabeza, 2002). This model reflects functional reorganization in the aging brain, compensating for declines in neural efficiency due to age-related structural and physiological deterioration. The second theory is the compensation-related utilization of neural circuits hypothesis (CRUNCH), which posits that under low cognitive load, older adults tend to exhibit overactivation or bilateral activation in the prefrontal cortex, whereas younger adults show more localized activation. As task demands increase, younger adults shift to a state of hyperactivation or bilateral activation to meet cognitive challenges, while older adults—already at their functional limits—experience cognitive decline accompanied by insufficient activation (Reuter-Lorenz and Cappell, 2008).

Hock et al. (1995) were the first to use fNIRS to examine age-related characteristics of hemodynamic changes in prefrontal cortex activation, finding that older adults exhibited significantly lower levels of oxyHb and total hemoglobin during cognitive tasks compared to younger adults. Aging is associated with reduced oxyHb in the brain, attenuated blood flow oscillations, and altered functional coupling between brain regions (Amiri et al., 2014; Tan et al., 2016; Wang et al., 2016). A substantial body of literature indicates that, at rest, older adults have lower prefrontal hemoglobin levels compared to younger individuals (Amiri et al., 2014; Hallacoglu et al., 2012; Tan et al., 2016), decreased connectivity between the left and right prefrontal cortices, increased connectivity between the prefrontal cortex and sensorimotor regions (Huo et al., 2018), and weakened neurovascular coupling with advancing age (Mukli et al., 2018). Studies on patients with MCI have found that older adults with MCI exhibit lower levels of oxygenated hemoglobin in the prefrontal cortex compared to cognitively normal older adults, with reduced oxyHb also observed in the bilateral frontal and temporoparietal regions (Viola et al., 2013). Studies using simultaneous recordings of fNIRS, ultrasound, and pulse oximetry have shown that individuals with MCI have lower cerebral blood flow and cerebral oxygen metabolism rates compared to healthy controls (Liu et al., 2014). These findings are consistent with positron emission tomography studies showing age-related declines in regional blood flow and glucose metabolism in the frontal lobes (Kalpouzos et al., 2009). Collectively, these studies suggest that cognitive aging is associated with changes in prefrontal activation and patterns of brain connectivity. With advancing age, neurovascular coupling weakens, blood vessels become stiffer and narrower, and white matter degeneration leads to reduced connectivity within the prefrontal cortex and between the prefrontal and motor cortices.

### Characteristics of cerebral oxygenation during executive function tasks in older adults

Executive function is a higher-order cognitive process and one of the most vulnerable to aging. It is responsible for monitoring and regulating other cognitive processes during the performance of complex cognitive tasks. Its essential role is to generate coordinated, organized, and goal-directed behavior (Etnier and Chang, 2009; Funahashi, 2001; Chen et al., 2011). Executive function pertains to the cognitive control processes that govern thought and behavior, exhibiting both a unified and multifaceted nature. While inhibitory control, working memory, and shifting are three distinct types of executive functions that are interconnected, they can still be differentiated from one another. These executive functions are among the psychological traits with the highest heritability estimates in behavioral genetics research (Friedman et al., 2008).

Numerous studies have confirmed that older adults exhibit lower cerebral oxygenation levels at rest compared to younger individuals; however, findings during task-related states—particularly during executive function tasks—have been inconsistent. In a verbal fluency task, Herrmann et al. (2006) found that the increase in oxyHb was higher in both hemispheres in younger adults than in older adults. Moreover, younger adults showed more pronounced left-hemispheric activation, a pattern not observed in older adults, who instead exhibited more symmetrical bilateral activation. Using high spatial resolution functional magnetic resonance imaging (fMRI), Ansado et al. (2013) also found significantly lower activation in the dorsolateral prefrontal cortex of older adults compared to younger individuals during a verbal fluency task. Schroeter et al. (2003) investigated age-related differences in brain activation during a Stroop task (a test of inhibitory control), focusing on the lateral prefrontal cortex, motor cortex, intraparietal sulcus, and primary visual cortex. Older adults showed poorer cognitive performance than younger adults, and fNIRS results indicated lower hemodynamic responses in the lateral prefrontal cortex in older participants. While younger adults showed increased lateral prefrontal activation during the incongruent (inhibitory) condition, older adults exhibited similar levels of activation in this region across both inhibitory and non-inhibitory conditions. Laguë-Beauvais et al. (2013) examined the functional neural networks involved in a Stroop task requiring both inhibition and switching, and found that prefrontal activation in older adults appeared to be more diffusely distributed compared to younger adults, as indicated by fNIRS data.

Similar findings were reported in fMRI studies; for example, Zysset et al. (2007) found that, under interference conditions of the Stroop task, older adults showed a greater increase in activation in the left dorsolateral prefrontal cortex and left inferior frontal gyrus compared to younger adults. Regarding working memory, studies have shown increased prefrontal activation (Agbangla et al., 2019) and more bilateral prefrontal engagement (Vermeij et al., 2012) in older adults during letter *n*-back tasks. One study found that older adults exhibited greater prefrontal activation than younger adults during spatial working memory maintenance and manipulation tasks (Yamanaka et al., 2014), whereas another study reported reduced activation in older adults under high-executive-demand spatial working memory conditions (Causse et al., 2019). Additionally, one study found that, compared to younger adults, older adults showed greater right dorsolateral prefrontal activation under low working memory load, but reduced activation in the same region under high working memory load (Wijeakumar et al., 2017).

Although the aforementioned findings are not entirely consistent, they generally support the CRUNCH. During executive function tasks, older adults tend to exhibit prefrontal overactivation in simpler tasks. This is attributed to limited cognitive resources in older adults, requiring the recruitment of additional neural resources to achieve performance levels comparable to those of younger adults. As a result, cortical activation becomes less lateralized or shows a bilateral pattern during cognitive tasks. However, at high cognitive loads exceeding functional capacity limits, further neural resource mobilization becomes unfeasible, resulting in regional deactivation. In summary, aging is associated with prefrontal overactivation under conditions of low cognitive load or executive demand, but with prefrontal underactivation under high cognitive load or executive demand.

### Characteristics of cerebral oxygenation changes associated with executive function during acute exercise in older adults

Dynamic fluctuations in cerebral hemoglobin concentrations occur during aerobic exercise (De Wachter et al., 2021). At 60% maximal oxygen uptake (VO₂max), cerebrovascular perfusion increases in prefrontal and motor cortices, concomitant with elevated oxyHb levels. When intensity exceeds this threshold, CBF reverts to baseline despite escalating metabolic demands (Querido and Sheel, 2007). This hemodynamic profile is consistent with the inverted U-shaped prefrontal lobe oxygenation–intensity relationship established by Rooks et al.’s (2010) systematic review, which peaked during moderate intensity aerobic exercise (40–60% VO_2_max).

Harada et al. (2009) demonstrated that during 5 min of brisk walking at 70% of the individual workload capacity, the changes in oxyHb concentration in the left prefrontal cortex and supplementary motor area were significantly greater than those observed during moderate-speed (50%) and slow-speed (30%) walking. Furthermore, older adults with lower gait ability exhibited higher levels of prefrontal activation, whereas those with higher gait ability showed relatively less prefrontal activation. Although different exercise intensities differentially affect cerebral oxyhaemoglobin, does the effect of varying exercise intensity on executive function differ? Ando et al. (2011) examined whether changes in cerebral oxygenation during different percentages of maximum oxygen uptake (VO_2_max) were associated with executive function performance during physical activity. Participants completed a Flanker task (a test of inhibitory control) while resting and at 40, 60, and 80% VO_2_max. The study found significant improvements in reaction time at rest and at 60% VO_2_max compared to rest, which was accompanied by increased cerebral oxyhaemoglobin without any statistical difference between these conditions. In contrast, cerebral oxyhaemoglobin decreased during 80% VO_2_max_,_ but cognitive performance did not significantly differ from the resting condition. Therefore, this study demonstrated that acute moderate-intensity exercise enhances executive function independent of changes in cerebral oxygenation and suggests that reduced brain oxygenation during high-intensity exercise may not impair executive function (Ando et al., 2011). A separate study reported similar results: during exercise at 70% VO₂max, cerebral hemoglobin concentration decreased, yet performance on the Stroop task improved (Lucas et al., 2012). Similarly, Ogoh et al. (2014) found that a 50-min graded cycling exercise significantly reduced Stroop reaction times, despite a concurrent gradual decrease in cerebral blood flow. This led them to propose that the improvement in cognitive function was attributable to neural activation in the brain rather than changes in cerebral blood flow.

Byun et al. (2016) found that even low-intensity acute exercise could improve executive function by enhancing prefrontal activation, which may be related to the increased systemic arousal level induced by exercise. According to Yerkes–Dodson law, an optimal level of arousal (often induced by exercise) can optimize the allocation of attention resources in the brain and thus enhance executive functions (Corbett, 2015). The increase in arousal levels is a key mediating variable through which exercise influences cognitive functions, especially those involving the prefrontal cortex. Belli et al. (2021) found that during both self-paced walking and brisk walking, older adults exhibited higher levels of prefrontal activation compared to younger adults. Furthermore, older adults showed an increase in the extent of activated prefrontal regions with increasing walking speed, a phenomenon not observed in younger adults. This suggests that older adults require greater prefrontal activation to maintain normal walking.

In summary, when exploring the mechanisms underlying the cognitive benefits of acute exercise, in addition to considering exercise intensity as a parameter, changes in arousal levels induced by exercise provide an important explanatory perspective. Different intensities of exercise may differentially affect neurotransmitter release and brain region activation by modulating arousal levels, ultimately leading to temporary improvements in executive function. Therefore, the arousal mechanism is an indispensable link in understanding the relationship between exercise intensity and cognitive effects (Eston, 2012).

### Characteristics of cerebral oxygenation changes associated with executive function after acute exercise in older adults

Endo et al. (2013) investigated the impact of acute exercise intensity/duration on post-exercise cognition while concurrently examining prefrontal oxyHb dynamics. Notably, 15-min ergometer exercise at 40% maximal voluntary contraction significantly improved Stroop performance alongside elevated prefrontal oxyHb concentrations. Soya et al. utilized fNIRS to compare young and elderly subjects, demonstrating sustained functional enhancement following exercise: 10 min of low-to-moderate intensity aerobic exercise improved accuracy on the Stroop test, accompanied by elevated levels of oxyHb in the prefrontal cortex (Byun et al., 2014; Hyodo et al., 2012; Yanagisawa et al., 2010).

Chinese researchers have made significant contributions to this domain. In a study conducted by Wen et al. (2015), a within-subject design was utilized wherein participants were randomly allocated to either an exercise or control condition to execute the Flanker task. The findings revealed that a 10-min session of moderate-intensity cycling significantly enhanced performance on the Flanker task. Furthermore, the concentration of oxyhemoglobin in the prefrontal cortex was notably higher in comparison to the control condition. Jiang et al. (2019) reported superior verbal fluency performance post-exercise in older adults, paired with bilateral prefrontal oxyHb elevation during cognitive tasks. Xu (2018) revealed that single Tai Chi sessions enhanced visuospatial memory while strengthening functional connectivity among task-relevant cortical areas. Our prior work further demonstrated that acute elastic band resistance exercise (low-moderate intensity) improved working memory in advanced-age adults, with increased oxyHb and decreased deoxyHb in dorsolateral prefrontal and frontopolar regions during cognitive tasks (Cai et al., 2023).

Based on the above discussion, most current studies have consistently shown an increase in cerebral oxyHb concentration following acute exercise. However, behavioral changes in cognitive function remain inconsistent. This discrepancy may be associated with factors such as cognitive task heterogeneity (complexity/type), participant characteristics (baseline age, cardiorespiratory fitness, cognitive status), and exercise protocol parameters (intensity, duration, modality).

### Characterization of chronic exercise-induced changes in cerebral oxygenation associated with executive function in older adults

Extensive research has confirmed that long-term regular physical exercise improves executive function in older adults. However, studies utilizing fNIRS technology to investigate the effects of long-term exercise interventions on cognitive function and its underlying neural mechanisms are relatively scarce, and their findings exhibit heterogeneity.

Wang et al. (2013) conducted a Tai Chi intervention study involving 12 older adults. Results showed a significant improvement in Stroop task scores post-intervention, but no significant changes were observed in prefrontal hemoglobin concentration during the task. Yang et al. (2019) similarly employed Tai Chi as an intervention, randomly assigning 26 older adults with no prior Tai Chi experience into two groups for an 8-week program. Compared to the control group, the Tai Chi group exhibited significantly shortened reaction times during the incongruent condition (high inhibitory demand) of the Flanker task post-intervention, along with a significant elevation in prefrontal oxyHb concentration during the task. Eggenberger et al. (2016) randomized 42 older adults into an exergame group and a balance training group for an 8-week intervention. Both interventions significantly reduced oxyHb concentration in the bilateral prefrontal cortex during accelerated walking (suggesting improved walking automaticity and reduced cognitive resource demand). At the end of the 30-s walking task, the exergame group showed a greater reduction in left prefrontal oxyHb concentration compared to the balance group. Crucially, exercise training-induced changes in prefrontal oxyHb (reflecting enhanced neural efficiency) were correlated with improvements in executive function. Coetsee and Terblanche (2017) examined the effect of exercise modality on oxyhemoglobin concentration during brain activation. The study involved 67 older adults who were randomly assigned to resistance training, high-intensity interval training (HIIT), moderate-intensity continuous training (MICT), or a control group. Following a 16-week intervention period, results indicated that both the MICT and the HIIT groups showed decreased brain activation during the Stroop task. Specifically, the MICT group had significantly higher deoxyhemoglobin levels in the naming and inhibition conditions post-intervention compared to baseline measurements. Both HIIT and MICT led to improved oxygenation efficiency during cortical activation over the 16 weeks, with no significant differences between these two forms of exercise. Guo (2020) conducted an 8-week intervention using the Flexi-bar exercise on middle-aged and elderly women, with sessions held three times per week for 40 min each at moderate intensity. The Flexi-bar is a portable training device based on resonance principles that stimulates systemic muscle groups through mechanical vibration to achieve fitness effects. The study found significant differences in activation levels between the left and right ventrolateral prefrontal cortex and the right frontal pole region during Stroop tasks after intervention. Cai et al. (2024) implemented a randomized controlled trial involving older adults, where the intervention group performed 16 weeks of low-to-moderate intensity resistance band exercises three times weekly for 40 min per session. The intervention protocol primarily included 10 training exercises targeting upper limb, lower limb, chest, and back muscles, with each exercise performed twice and each repetition consisting of 10–12 cycles. Results demonstrated that regular low-to-moderate intensity resistance band exercises could improve working memory performance in older adults; while prefrontal activation was not significant under low memory load conditions, bilateral ventrolateral prefrontal cortex, left dorsolateral prefrontal cortex, and left frontal pole regions showed significant activation under medium and high memory load conditions.

In summary, the incorporated literature consistently demonstrates that long-term physical exercise improves behavioral performance on executive function tasks. However, the hemodynamic results measured by fNIRS (e.g., task-related oxyHb concentration changes) are inconsistent. Key factors potentially contributing to these discrepancies include: study design (e.g., sample size, control group setup), primary research focus, heterogeneity in the enrolled population (age, health status, baseline cognition and fitness), intervention protocol (exercise type, intensity, frequency, duration, adherence), fNIRS measurement parameters (e.g., optode montage, cortical coverage, channel selection, signal processing pipeline), and differences in device manufacturers and models.

Based on current evidence, this review argues that a unidirectional change (increase or decrease) in a single task-related hemodynamic metric (e.g., the amplitude of oxyHb concentration change) cannot be simplistically interpreted as indicating cognitive improvement or decline. Its significance requires comprehensive interpretation considering the specific study design (particularly whether behavioral improvement was observed), the nature of the cognitive task, the brain regions examined, and the neural efficiency theory (where reduced activation often signifies enhanced efficiency).

## Discussion

### Dual-phase neural compensation model

This study systematically synthesizes evidence from fNIRS applications in exercise-induced executive function improvements in older adults. Findings demonstrate that acute and chronic exercise enhance executive function through distinct neural mechanisms. We innovatively propose the “dual-phase neural compensation model” (Figure 2) (Calverley et al., 2020), which integrates the temporal dynamics of exercise-induced brain functional adaptations and reconciles the apparent contradiction between “prefrontal activation enhancement” and “activation reduction” observed in prior studies.

In the acute phase, the brain responds to the novel physiological stressor of exercise by expanding its neural resource pool, primarily reflected as increased prefrontal activation (or cerebral oxygenation) in fNIRS signals. In contrast, the chronic phase is not an isolated phenomenon but is built upon repeated acute bouts of exercise. Over time, these repeated acute stressors trigger a cascade of molecular and cellular adaptations—such as increased synaptic plasticity, angiogenesis, and neurogenesis—that fundamentally alter the brain’s functional architecture. As a result, the brain transitions from a state of “resource expansion” to one of “neural efficiency optimization,” where it can achieve the same or better cognitive performance with reduced metabolic demand.

To resolve the apparent paradox of cerebral oxygenation responses—some studies showing increased activation and others showing reductions—we suggest that these divergent findings are likely due to complex neurotransmitter interactions within the neurovascular unit. Depending on whether excitatory (e.g., glutamate) or inhibitory (e.g., GABA) synapses are preferentially engaged, the resulting vascular response can be markedly different. During acute high-intensity exercise, excitatory neurotransmission may dominate, leading to increased cerebral blood flow and oxygenation. In contrast, during chronic stages where neural networks become more efficient and may rely more heavily on inhibitory control to fine-tune processing, the neurovascular response may manifest as a relative reduction in cerebral oxygenation despite improved cognitive performance. This nuanced understanding is consistent with the “dual-phase neural compensation model,” where both phases are interdependent and mediated by distinct neurovascular dynamics.

### Neural resource pool expansion

Acute exercise expands prefrontal neural resources through the release of a variety of neurotransmitters and neurotrophic factors (e.g., catecholamines, BDNF, IGF-1). This manifests as significantly elevated hemodynamic activity in the dorsolateral prefrontal cortex, inducing transient cognitive facilitation with immediate improvements in inhibitory control and working memory task performance (Chang et al., 2012; Kamijo et al., 2004). In addition to catecholamines, the acute phase also involves increased glutamatergic neurotransmission (Baskerville et al., 2023), which together facilitates neural activation in response to heightened arousal stimuli. While the underlying neurophysiological mechanisms remain unclear, these mediators collectively enhance prefrontal cortex activation.

### Neural efficiency optimization

Chronic exercise induces structural neuroplasticity (including anterior cingulate cortex gray matter volume expansion and white matter integrity enhancement) that optimizes neural resource allocation (Erickson et al., 2011). The hallmark characteristic is significantly reduced prefrontal activation intensity during identical cognitive tasks (e.g., Coetsee and Terblanche, 2017) reported a 22.3% decrease in oxyHb amplitude during Stroop performance after moderate-intensity continuous training, reflecting fundamental improvements in neural information processing efficiency.

### Moderating effects of exercise parameters

Intensity-dependent effects: Prefrontal oxygenation exhibits an inverted U-shaped relationship with exercise intensity (Rooks et al., 2010). Moderate intensity (40–60% VO₂max) optimally enhances neurovascular coupling, whereas excessive intensity (>80% VO₂max) may diminish cognitive gains due to metabolic overload (Ando et al., 2011).

Modality specificity: Aerobic exercise (e.g., brisk walking) primarily strengthens frontoparietal functional connectivity (Byun et al., 2014); resistance training improves working memory via DLPFC efficiency gains (Cai et al., 2024); coordinative exercise (e.g., Tai Chi) enhances cognitive flexibility through cerebellar-prefrontal pathway reinforcement (Yang et al., 2019).

### Limitations

Although this study synthesizes neural mechanisms underlying exercise-induced improvements in executive function among older adults through fNIRS evidence, it is important to acknowledge that our conclusions are constrained by the inherent technical characteristics of the primary methodology relied upon. While fNIRS offers unique advantages for exercise research, its spatial resolution is relatively limited, and photon penetration depth is primarily confined to the cerebral cortex. This leads to two major limitations in the current body of evidence: first, a difficulty in precisely dissociating activation patterns between adjacent functional subregions within the prefrontal cortex (e.g., dorsolateral vs. ventrolateral prefrontal cortex); second, a near inability to directly detect activity changes in deep brain structures critical for learning and memory consolidation (e.g., hippocampus, basal ganglia) in response to exercise interventions. Consequently, the neural activation patterns described in this review predominantly reflect cortical-level hemodynamic responses and do not encompass the complete “cortical–subcortical” neural networks involved in exercise-modulated cognition. Future research must employ multimodal neuroimaging approaches to bridge these gaps and construct a more comprehensive theoretical model.

## Conclusions and future perspectives

The findings demonstrate that brain aging is closely associated with alterations in prefrontal cortex activation patterns and functional brain network connectivity during both resting and task states. At rest, aging is manifested by reduced cerebral oxygenation, diminished blood flow oscillations, and aberrant functional coupling across brain regions. During executive function tasks, older adults frequently compensate for declines in core brain regions by recruiting additional neural resources, including non-task-specific areas. This compensation manifests as overactivation/ bilateral activation under low cognitive load and insufficient activation under high cognitive load, aligning with the CRUNCH (Reuter-Lorenz and Cappell, 2008).

Exercise-induced enhancements in executive function exhibit stage-dependent characteristics: acute exercise is predominantly governed by “neural resource pool expansion”, while chronic exercise is centrally characterized by “neural efficiency optimization” through distinct neurobiological pathways.

fNIRS technology holds irreplaceable value in exercise-cognitive neuroscience research due to its unique advantages: strong resistance to electromagnetic interference, high motion robustness, good ecological validity, and portability. Currently, most related studies employ cross-sectional designs or small-sample intervention trials, with a relative scarcity of high-quality randomized controlled trials (RCTs). Future research urgently needs to:

1. Conduct rigorous large-sample RCTs: Design and implement methodologically sound, large-scale RCTs to rigorously validate the specific effects of different types, intensities, and durations of physical exercise programs on hemodynamic characteristics (activation patterns, brain network connectivity) within the prefrontal cortex during executive function tasks in older adults.
2. Leverage fNIRS motion tolerance: Utilize fNIRS’s high tolerance for motion artifacts to enable real-time monitoring of hemodynamic dynamics in key brain regions (e.g., prefrontal cortex) during physical exercise itself (not merely pre/post-exercise). This will provide objective evidence for understanding the immediate neural mechanisms of exercise-cognition interactions and inform the development of precise exercise prescriptions.
3. Implement multimodal neuroimaging: Conduct multimodal brain imaging studies to overcome fNIRS’s limitation in penetration depth (primarily restricted to the cerebral cortex). Combining fNIRS with electroencephalography/event-related potentials (EEG/ERP) for high temporal resolution and functional magnetic resonance imaging (fMRI) for high spatial resolution and whole-brain coverage will provide complementary neuroelectrophysiological and deep-brain/whole-network information. This integrated approach will offer more robust evidence for comprehensively elucidating exercise-induced neural circuit remodeling and brain network reorganization.
